# Looking into the crystal ball: quality of life, delinquency, and problems experienced by young male adults after discharge from a secure residential care setting in the Netherlands

**DOI:** 10.1186/s13034-019-0305-0

**Published:** 2019-11-18

**Authors:** E. A. W. Janssen-de Ruijter, E. A. Mulder, I. L. Bongers, L. Omlo, Ch. van Nieuwenhuizen

**Affiliations:** 1 GGzE Centre for Child & Adolescent Psychiatry, PO BOX 909 (DP 8001), 5600 AX Eindhoven, The Netherlands; 2 Tilburg University, Scientific Center for Care & Wellbeing (Tranzo), Tilburg, The Netherlands; 3 Leiden University Medical Center, Leiden, The Netherlands; 4 Intermetzo-Pluryn, Nijmegen, The Netherlands

**Keywords:** Follow-up, Young adulthood, Quality of life, Delinquency, Residential care, Risk profiles, Self-report

## Abstract

**Background:**

Adolescents in residential care are a vulnerable population with many problems in several life areas. For most of these adolescents, these problems persist after discharge and into adulthood. Since an accumulation of risk factors in multiple domains increases the likelihood of future adverse outcomes, it would be valuable to investigate whether there are differences in life after residential care between subgroups based on multiple co-occurring risk factors.

**Aims and hypothesis:**

The aim of this exploratory follow-up study is to explore differences between young adults—classified in four risk profiles—in relation to life after discharge from a secure residential care setting. It is hypothesised that young adults with a profile with many risks in multiple domains will experience more problems after discharge, such as (persistent) delinquency, compared to young adults with a profile with lower risks.

**Methods:**

Follow-up data were collected from 46 former patients of a hospital for youth forensic psychiatry and orthopsychiatry in the Netherlands. In order to illustrate these young adults’ life after discharge, self-reported outcome measures divided into five domains (i.e., quality of life, daily life, social life, problems, and delinquency) were used. Differences between four classes based on pre-admission risk factors, which were identified in a previous study by latent class analysis, were explored by three (non-)parametric statistical tests.

**Results:**

Life after discharge for most young adults was characterised by close friends and a high quality of life, but also by substance abuse, professional support, debts, and delinquency. Only a few significant differences between the classes were found, primarily between young adults with risk factors in the individual, family, school, and peer domains and young adults in the other three classes.

**Conclusions:**

Young adults experience a high quality of life after discharge from secure residential care, despite the presence of persistent problems. Some indications have been found that young adults with risk factors in four domains are at greatest risk for persistent problems in young adulthood. Because of the high amount of persistent problems, residential treatment and aftercare should focus more on patients’ long-term needs.

## Background

Adolescents in residential care are a vulnerable population with many problems in several life areas [1, 2]. Usually, these adolescents have had to deal with various adverse circumstances from an early age, for which they have often had a rich history of provided care before they were finally admitted to residential care [3–6]. For most of these adolescents, these problems even persist in their lives after discharge from residential care and into adulthood [7, 8]. Examples of such persistent problems occur in education [9], employment [3, 8, 9], mental health [8, 9], delinquency [9], financial problems [8], problematic alcohol and drug use [3, 8], and unstable relationships [8]. These persistent problems seem to indicate that residential treatment is not sufficient for everyone.

Risk factors play an important role in the prediction of persistent problems, such as delinquency [10, 11]. Understanding how risk factors relate to the persistence of problems remains an important challenge to improve the effectiveness of residential treatment. Some studies have demonstrated that specific risk factors are related to problematic life outcomes, such as early age at first conviction to persistent delinquency [7, 12], history of maltreatment to more serious delinquency [9], more hospitalisations to future mental health problems [9], and substance use to conduct problems, delinquency, and deterioration of symptoms [7, 13]. However, most adolescents admitted to residential care are subject to not one but multiple risk factors [6, 14]. Since exposure to an accumulation of risk factors in multiple domains increases the likelihood of future adverse outcomes [15], a focus on co-occurring risk factors could add to our understanding of the population of adolescents who are admitted to residential care.

Few studies have investigated whether subgroups with multiple co-occurring risk factors differ on future outcomes [14, 16]. In a study among childhood first-time arrestees, it was demonstrated that children who displayed high levels of internalizing, externalizing, peer and family problems were most likely to show future antisocial behaviour [16]. In addition, in a study among adolescents with psychiatric problems, it was found that children with multiple needs run the greatest risk for adverse outcomes, such as involvement with the juvenile justice system [14]. The findings of these studies, i.e., that groups of children with multiple risk factors experienced the greatest risk for adverse outcomes later in life, underscore the added value of investigating future outcomes for separate subgroups with multiple co-occurring risk factors.

Adolescents in residential care, with multiple risk factors in various domains [17, 18], are at substantial risk for long-term delinquency and other problems. Identifying homogeneous subgroups in this population may enhance insight into which young adults will experience major problems in young adulthood. In a previous study on the same population as in the present paper, Janssen-de Ruijter et al. [18] identified four classes based on prominent risk factors for (persistent) disruptive behaviour and delinquency: (1) adolescents with multiple risks in the individual, peer, and school domains (Class 1); (2) adolescents with various risk factors in the individual, family, peer, and school domains (Class 2); (3) adolescents with risks primarily in the peer domain (Class 3); and (4) adolescents who experienced primarily risks in the family domain (Class 4). Additional analyses demonstrated that adolescents in the two classes with a profile with higher risks in more domains (Classes 1 and 2), which primarily differed on their family risks, had more often committed multiple offences before admission than adolescents in the other two classes with a profile with lower risks [18]. Given this reported difference in previous delinquent behaviour and in (the amount of) co-occurring risk factors, these classes of adolescents admitted to secure residential care may also differ in their risks of long-term delinquency and other adverse problems after residential care.

Even though earlier studies have identified persistent problems of young people after residential care, less is known about how they experience the diverse aspects of their own lives. In a study on the experiences of adolescents who have left secure residential care, approximately all adolescents reported experiencing problems [8]. However, despite these problems, quality of life in most life domains was generally reported as high [8]. This reported high quality of life corresponds with the findings of another study among another sample of adolescents after discharge from secure residential care [19]. More specifically, the findings of both studies showed that the adolescents are most satisfied with their safety and least satisfied with their finances [8, 19]. Another finding from the study on the experiences of young people after residential care is that 1 year after discharge, the majority of adolescents reported that they are involved in structured activities such as work or education [8].

Thus, previous follow-up studies have demonstrated both persistent problems and a primarily high quality of life among young adults in their lives after residential care [e.g., 8, 12]. In an attempt to search for possible explanations for young adults who experience more or fewer problems in adulthood, earlier studies of specific populations demonstrated that subgroups with many co-occurring risk factors have the greatest risk for negative life outcomes [14, 16]. The aim of this exploratory follow-up study is to explore differences between young adults—classified in four previously found risk profiles [18]—with regard to their quality of life, daily life, social life, delinquency, and other problems after discharge from a secure residential care setting. Based on the findings of previous follow-up studies, it is hypothesised that young adults with profiles with higher risks in multiple domains and with a history of serious delinquency, disruptive behaviour, and substance abuse (Classes 1 and 2) will experience more problems after discharge than young adults with profiles with lower risks [14, 16]. Since no research is known that has investigated the relationship between risk profiles and quality of life, no hypotheses can be formulated for quality of life.

## Methods

### Setting

All participants were former male patients of the Catamaran, a hospital for youth forensic psychiatry and orthopsychiatry in the Netherlands. This secure residential care setting offers intensive multidisciplinary treatment to adolescents and young adults aged between 14 and 23 years. Adolescents and young adults admitted to this setting have been sentenced under Dutch juvenile criminal law, Dutch juvenile civil law, or are admitted voluntarily. Measures under Dutch juvenile criminal law are aimed at treatment and rehabilitation of adolescents and young adults who have committed serious offences. Measures under Dutch juvenile civil law are applied to adolescents whose development is at risk and whose parents or caregivers are not capable of providing the required care. Irrespective of the type of measure, all adolescents and young adults admitted to this hospital display multiple severe problems in several areas of their lives and suffer from major psychiatric problems and/or severe behavioural problems. Furthermore, many of them have engaged in delinquent behaviour.

### Sample

The sample consisted of 46 young men who had been discharged from the hospital between April 2009 and August 2013. Before admission, five participants were living with one or both of their parents. The other participants were living in detention centres (two participants), juvenile justice institutions (23 participants), or in residential/crisis care (16 participants). All participants but one had had previous contact with mental health services before admission to the hospital. The majority of the sample (38 participants) was convicted of one or more offences before admission.

Half of the sample (23 participants) completed treatment before discharge (i.e., completers). For the other half of the participants, treatment was terminated prematurely: eight participants terminated treatment against the advice of the clinician, six participants were expelled and nine participants were, in accordance with the clinician, transferred to another care setting before their treatment goals were achieved and treatment was completed. The majority of the sample (34 participants) had some form of aftercare immediately after discharge. After discharge, most completers went home (ten participants) or to sheltered housing (nine participants). Less common discharge settings among the completers were residential care (three participants) and independent living (one participant). Among the non-completers, the most common discharge setting was also home (nine participants). Other discharge settings were juvenile justice institutions (four participants), residential care settings (three participants), independent living (three participants), and other settings (two participants). For two non-completers, the discharge setting was unknown, since they ran away from the hospital to an unknown place.

### Risk profiles

The 46 young men participating in this study were part of a sample of 270 patients in a previous study in which four risk profiles were identified by latent class analysis [LCA; 18]. LCA uses categorical latent variables to explain relationships among observed variables, which results in the identification of classes of individuals with similar characteristics [20]. In the previous study, eleven co-occurring risk factors in individual, family, peer, and school domains which were present at the time of admission to the hospital were used. Items of the Structured Assessment of Violence Risk in Youth [SAVRY; 21] and the Juvenile Forensic Profile [JFP; 22] were used to operationalise the eleven risk factors. The individual domain contained three risk factors: hyperactivity, cognitive impairment, and history of drug abuse. The family domain consisted of three risk factors: exposure to violence in the home, physical/emotional abuse, and criminal behaviour of family members. The three risk factors in the peer domain were peer rejection, involvement in criminal environment, and lack of secondary network. The school domain comprised two risk factors: low academic achievement and truancy.

**Fig. 1:**
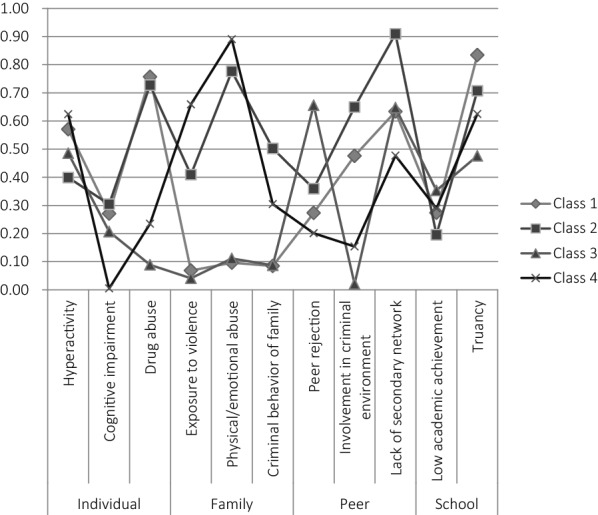
Four-class solution (N = 270; 18)

Based on fit indices, the four-class solution (see Fig. 1) best fit the data. Class 1 (*n* = 119) represented adolescents with risk factors in three domains; i.e., the individual (drug abuse), peer (involvement in criminal environment), and school (truancy) domains. Adolescents in Class 2 (*n *= 70) had risk factors in all four domains, such as drug abuse in the individual domain, physical/emotional abuse in the family domain, involvement in criminal environment in the peer domain, and truancy in the school domain. Class 3 (*n *= 49) had the lowest risks overall, yet they had the highest risk for peer rejection compared to the adolescents in the other classes. Finally, Class 4 (*n *= 32) represented adolescents with risk factors primarily in the family domain (e.g., physical/emotional abuse and exposure to violence in the home). Characteristics of adolescents in Classes 1 and 2 were rather similar, for example substance use and delinquent behaviour before admission were both common in adolescents in these classes. The main difference between these two classes was the high number of family risk factors in Class 2. The adolescents in Classes 3 and 4 had distinctive characteristics, such as the highest prevalence of autism spectrum disorders and sex offences in Class 3, and the highest percentage of no previous convictions in Class 4.

### Procedure

**Fig. 2:**
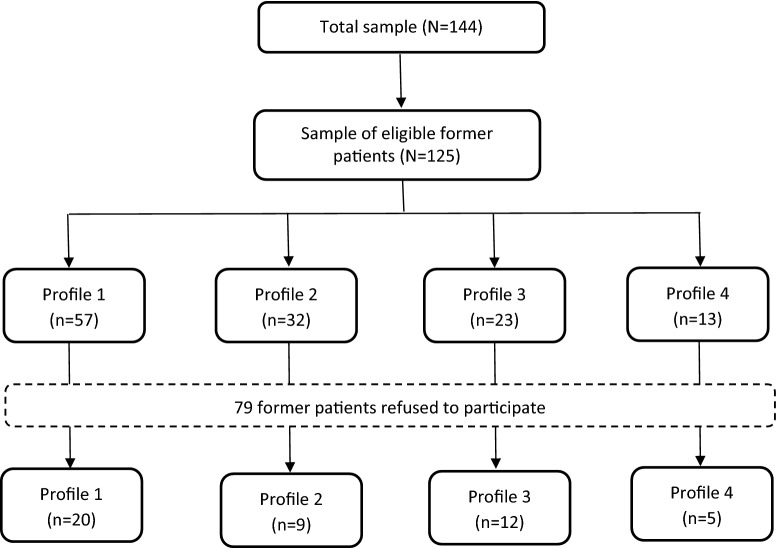
Flowchart FU-study

Inclusion criteria were: (1) being 18 years or older at the time of the exploratory follow-up study, and (2) admitted between April 2005 and October 2013 with a minimum stay of 3 months. Patients discharged before April 2009 were excluded, because information about these patients had not been transferred to the digital patient database introduced in April 2009. Of all former patients, 144 fulfilled these inclusion criteria. Seventeen former patients could not be reached at the time of follow-up, despite extensive searches, and two patients were deceased. Therefore, the eligible sample consisted of 125 male former patients of which 46 (37%) were included (see Fig. 2). The other 79 former patients refused to participate for the following reasons: lack of time (five persons), because they did not want to think back on their experience in care (13 persons), because they did not feel like it (24 persons), and because there was no financial reward (two persons). The remaining 35 former patients gave no reason for refusal. Differences between the included sample (*n* = 46) and excluded sample (*n* = 79) were investigated for the following background and discharge variables: length of stay at the hospital, time after discharge, age at the time of the follow-up study (FU-study), ethnicity, the absence of previous convictions, early onset of problem behaviour, discharge placement, completer, and classifications at discharge. Having an attention deficit/hyperactivity disorder at discharge was the only significant difference between the included sample (39%) and the excluded sample (19%; *F*(1, 143) = 6.595, *p* = .011).

Of the 46 participants, twenty participants were classified in Class 1, nine participants in Class 2, 12 in Class 3, and five participants were classified in Class 4. No significant differences in the participation rates of the four classes between the eligible sample of 125 former patients and the included sample of 46 former patients were found.

At least 1 year after discharge from the hospital, all former patients who matched the inclusion criteria were sent a letter which explained the aim of the study. In addition, the letter contained a notification that the researcher was going to contact the former patient 1 week later. In this phone call, the researcher was able to clarify, if necessary, the goal of the FU-study and could ask the former patient for his willingness to participate. If the former patient could not be reached by phone, a second letter was sent with a reply card and envelope. On the reply card, the former patient could fill in whether he wanted to engage in the study or not and he was asked for his telephone number in case he wished to participate. The letter also contained the researcher’s telephone number and e-mail address to allow the former patient to contact the researcher via telephone, WhatsApp, or e-mail. In cases where no address and only a telephone number was retrieved, the researcher called the former patient to briefly explain the study. Afterwards, the researcher asked for his permission to send an information letter. If the former patient immediately declared that he did not wish to participate, he was not contacted again. In cases where no contact information at all could be retrieved, an Internet search was conducted in order to find a way to contact the former patient; for instance, by means of social media. The recruitment of participants was carried out by one researcher.

The FU-study consisted of questionnaires and a structured interview, and was conducted at a public location, the participant’s home, or a(n) (judicial) institution. The interviews for the FU-study were, after a short training, conducted by two researchers and a trainee. The interviewers took extensive notes during the interviews in the presence of the participants. Before the interview, a verbal and written explanation of the study was once again provided and participants were fully assured of their anonymity. Written informed consent was obtained from each participant. In total, completion of the questionnaires and the interview took about 1.5 h.

The proposal of the FU-study was submitted to the institutional review board (IRB) of GGzE, the Institute of Mental Health Care. On 15 January 2013, the IRB concluded that this study was in accordance with the prevailing medical ethics in the Netherlands. In addition, they declared that the study did not fit the conditions of the Medical Research Involving Human Subjects Act and, therefore, that no additional examination by a medical ethical committee was required for this study.

### Instruments

To outline the young adults’ life after residential care, a large number of variables was used and these were divided into five categories; i.e., quality of life, daily life, social life, problems, and delinquency. These variables were operationalised based on the following questionnaires and the interview from the FU-study (see Table 1).

**Table 1:** Operationalisation of the measurements

Domain	Variable	Instrument	Question	Scores
Quality of life	Quality of life	MANSA	12 subjective questions Total mean score	0 = low to average scores (scores 4 or lower) 1 = high scores (scores higher than 4)
Daily life	Living situation	MANSA	With whom do you live?	0 = independent living (alone, with a partner, with peers) 1 = living with (foster) family (with own parents, with foster parents, with another family) 2 = residential care facilities (judicial institutions, sheltered housing, psychiatric hospitals, residential care)
	Structured activities	MANSA	What is your work situation?	0 = no structured activities (unemployment, work in prison, intention of new studies in the future) 1 = structured activities (education, work, sheltered employment, volunteer work)
	Social security benefits	MANSA	Do you receive social security benefits?	0 = no social security benefits 1 = social security benefits
Social life	Intimate relationship at the time of the FU-study	Interview	Do you have a relationship at this time?	0 = no 1 = yes
	Intimate relationship after discharge	Interview	Have you had (other) relationships since your discharge from the hospital?	0 = no 1 = yes
	Number of close friends	ASR	Approximately how many close friends do you have? (Do not include family members)	0 = none 1 = one to three 2 = four or more
	Delinquent peers	Interview	Did one of your friends have contact with police or justice authorities in the past year?	0 = no 1 = yes
	Quality relationship with mother	ASR	Compared with others, how well do you get along with your mother?	0 = worse than average 1 = average 2 = better than average
	Quality relationship with father	ASR	Compared with others, how well do you get along with your father?	0 = worse than average 1 = average 2 = better than average
Problems	Problem behaviour	ASR	Internalising and externalising syndrome scales	0 = no problems (raw scores in the normal range) 1 = problems (raw scores in the borderline or clinical range)
	Debts	Interview	Do you have debts at this moment?	0 = no 1 = yes
	Substance abuse	Substance use questionnaire	On how many weekdays (Monday to Thursday) do you usually drink alcohol? On how many of the weekend days (Friday to Sunday) do you usually drink alcohol? How often have you used cannabis (marijuana) or hash in the last 12 months? How often have you used cocaine (coke or white) or heroin (horse, smack, or brown) in the past 12 months? How often have you used XTC (ecstasy, MDMA), magic mushrooms, amphetamines (uppers, pep, or speed), or GHB in the past 12 months?	0 = no (soft drug and alcohol use less than 4 days a week, and hard drug use less than 2 days a week) 1 = yes (soft drug or alcohol use at least 4 days a week, and/or hard drug use more than 2 days a week) 999 = missing (alcohol, soft drug and/or hard drug use missing and the other variable(s) scored no)
	Professional support	Interview	Do you receive any professional support at this time?	0 = no 1 = yes
Delinquency	Offences after discharge	Interview	Have you committed one or more offences after discharge for which you were or were not convicted, or which are unknown to the police?	0 = no 1 = yes
	Violent offences^a^ after discharge	Interview	If yes, which type of offence(s) did you commit?	0 = no violent offences 1 = one or more violent offences
	Non-violent offences^a^ after discharge	Interview	If yes, which type of offence(s) did you commit?	0 = no non-violent offences 1 = one or more non-violent offences

^a^The difference between violent and non-violent offences was based on the definition of violence in the Structured Assessment of Violence Risk in Youth (SAVRY): “Violence is a deed of abuse or physical violence sufficient to cause an injury to one or more persons (for instance, cuts, bruises, bone fractures, death, et cetera), no matter whether this injury really occurred or not; every form of sexual assault; or threat with a weapon. In general, these deeds need to be sufficiently serious to (could) have led to prosecution for criminality.” [21]

The *Manchester Short Assessment of Quality of Life* [MANSA; 23] consists of demographic items and 12 subjective questions. The subjective questions cover satisfaction with, for example, financial situation, leisure activities, and personal safety. The questions were rated on a 7-point Likert scale, ranging from 1 (couldn’t be worse) to 7 (couldn’t be better). The Dutch manual of the MANSA describes good reliability and validity for several populations including patients with severe psychiatric problems [23]. In this study, Cronbach’s alpha of the 12 subjective questions was .82.

The *Adult Self Report* (ASR) is a self-report questionnaire for adults aged 18 to 59 [24] that measures behaviour in the last 6 months. The list consists of two broad band scales: internalising and externalising problem behaviour. In the list, all items were scored on a 3-point Likert scale: 0 = not true, 1 = somewhat or sometimes true, and 2 = very true or often true. Scores on the broad band scales can be categorised into three ranges: normal range, borderline range, and clinical range. In this study, Cronbach’s alpha of the internalising broad band scale was .93 and Cronbach’s alpha of the externalising broad band scale was .89.

The *Substance Use Questionnaire* was derived from the *Juvenile Crime Monitor* (JCM) of the WODC, Ministry of Security and Justice in the Netherlands [25]. The substance use questionnaire consists of ten questions about alcohol and drug use; e.g., on how many weekdays (Monday to Thursday) do you usually drink alcohol?

The *Follow*-*Up Interview* is a structured interview with 17 primarily closed-ended questions, which explore remaining issues about daily life, social network, delinquency, and professional support. Examples of questions were whether the participant had any debts and whether the participant received any professional support at that time.

### Statistics

First, a skewness–kurtosis test in SPSS 19.0 (Statistical Packages for the Social Sciences 19.0 for Windows, 2010) was used to determine normality of the dependent variables. Second, to determine the significance (*p *< .05) of the encountered differences between the four classes, three (non-)parametric statistical tests were conducted. The Fisher’s exact test was conducted for nominal dependent variables. For ordinal dependent variables and non-normally distributed continuous dependent variables, the Kruskal–Wallis one-way analysis of variance was conducted. For normally distributed continuous dependent variables, analysis of variance (ANOVA) was conducted with Bonferroni correction to correct for multiple testing. While the three (non-)parametric statistical tests point at overall significant differences between the four classes, class-specific adjusted residuals were used to see where the differences occur. An adjusted residual above 1.96 or below − 1.96 indicates the value in a specific class is, respectively, larger or smaller than the values of the other classes. Significance tests are primarily used to eliminate variables of lesser interest. Therefore, the alpha level was not adjusted for multiple testing (e.g., using a Bonferroni correction) because much stricter alpha levels would potentially hide possibly interesting correlates of the encountered classes.

## Results

### Sample description

The total group had an average age of 21.9 (range = 18–27) at the time of the FU-study and their average time after discharge was approximately 3 years with a range of 1 to 6 years after discharge. With regard to their stay at the hospital, the average length of stay was 20.2 months and approximately half of the patients were sentenced under Dutch juvenile criminal law (46%). The average age at admission was 16.8 (range = 14–21). The majority of the patients (83%) was convicted of one or more offences before admission and 59% of the total group had an early onset of problem behaviour (before age 12). After discharge, most patients (77%) went to a less restrictive place (e.g., to family or sheltered housing). More sample characteristics are displayed in Table 2.

**Table 2:** Sample description (N = 46)

	Total group (N = 46)	Class 1 (*n* = 20)	Class 2 (*n* = 9)	Class 3 (*n* = 12)	Class 4 (*n* = 5)	*Χ*^2^/F	*p*-value
	M (SD)	M (SD)	M (SD)	M (SD)	M (SD)		
Length of stay at the hospital (in months)	20.2 (11.8)	19.6 (11.4)	26.1 (16.7)	19.1 (7.9)	14.8 (10.2)	F = 1.169	.333
Time after discharge (in months)	39.2 (16.7)	39.7 (18.2)	38.2 (17.5)	35.4 (13.2)	47.6 (18.7)	*Χ*^2^ = 2.640	.451
Age at admission	16.8 (1.6)	16.4 (1.3)	18.1 (2.0)	16.7 (1.7)	16.8 (.8)	*Χ*^2^ = 5.321	.150
Age at the time of the FU-study	21.9 (2.4)	21.4 (1.9)	23.7 (3.2)	21.3 (2.2)	22.0 (2.4)	F = 2.393	.082

	*n* (%)	*n* (%)	*n* (%)	*n* (%)	*n* (%)		
Judicial measure						*Χ*^2^ = 9.784	.084
Criminal law	21 (46%)	5 (25%)↓	7 (78%)↑	7 (58%)	2 (40%)		
Civil law	21 (46%)	13 (65%)↑	2 (22%)	3 (25%)	3 (60%)		
Voluntary	4 (9%)	2 (10%)	0 (0%)	2 (17%)	0 (0%)		
Immigrants (1st or 2nd generation)^a^ (*n* = 41)	15 (37%)	6 (33%)	6 (67%)↑	1 (11%)	2 (40%)	*Χ*^2^ = 5.916	.104
No previous convictions	8 (17%)	3 (15%)	1 (11%)	3 (25%)	1 (20%)	*Χ*^2^ = 1.130	.849
Early onset of problem behaviour (< 12 years)	27 (59%)	9 (45%)	7 (78%)	7 (58%)	4 (80%)	*Χ*^2^ = 3.591	.319
Psychopathology at discharge^b^							
Disruptive behaviour disorder	17 (37%)	7 (35%)	6 (67%)↑	4 (33%)	0 (0%)	*Χ*^2^ = 5.992	.103
Autism spectrum disorder	20 (44%)	10 (50%)	1 (11%)↓	9 (75%)↑	0 (0%)↓	*Χ*^2^ = 12.513	.004
Attention deficit/hyperactivity disorder	18 (39%)	10 (50%)	1 (11%)	5 (42%)	2 (40%)	*Χ*^2^ = 4.064	.269
Substance disorder	11 (24%)	7 (35%)	4 (44%)	0 (0%)↓	0 (0%)	*Χ*^2^ = 8.579	.022
Reactive attachment disorder	10 (22%)	2 (10%)	5 (56%)↑	0 (0%)↓	3 (60%)↑	*Χ*^2^ = 13.826	.001
Less restrictive discharge placement^c^ (*n* = 44)	34 (77%)	17 (85%)	5 (63%)	8 (73%)	4 (80%)	*Χ*^2^ = 2.111	.615
Completer^d^	23 (50%)	9 (45%)	1 (11%)↓	10 (83%)↑	3 (60%)	*Χ*^2^ = 11.223	.008

All information in this table is derived from the electronic patient database of the hospital

↑Adjusted residual > 1.96: higher value than expected; ↓Adjusted residual < − 1.96: lower value than expected

^a^1st and 2nd generation immigrants were operationalised as persons who were born abroad themselves and persons with at least one parent who was born abroad

^b^Psychopathology at discharge is derived from the, at the time of discharge, most recent DSM-IV-classifications from the patient database

^c^A less restrictive discharge placement was operationalised as a discharge to home, other family or friends, sheltered housing, independent living, homeless, or foster care

^d^Completer was operationalised as a completed treatment in which all treatment goals were achieved

Differences between the four classes were found in psychopathology at discharge (autism spectrum disorder: *Χ*^2^ = 12.513, *p *= .004, substance disorder: *Χ*^2^ = 8.579, *p *= .022, reactive attachment disorder: *Χ*^2^ = 13.826, *p *= .001) and in completers (*Χ*^2^ = 11.223, *p* = .008). At discharge, most young adults in Class 3 (75%) were classified with autism spectrum disorder. Substance disorders were only classified in young adults in Classes 1 and 2. Reactive attachment disorders were most classified in young adults in Classes 2 and 4. Toward completed treatment at discharge, the majority of the young adults in Class 3 (83%) were completers, whereas the majority of the young adults in Class 2 (89%) terminated treatment prematurely.

### Quality of life

In the total group, approximately all young adults (87%) reported a high quality of life at the time of the FU-study, measured by the mean score of the twelve questions of the MANSA (see Table 3). The majority of the young adults also reported high scores on most separate questions; e.g., on the number and quality of friendships, leisure activities, personal safety, and physical and mental health. On life as a whole, job situation, and financial situation, young adults less often reported a high score (44–54%).

**Table 3:** Quality of life after discharge (N = 46)

	Total group (N = 46)	Class 1 (*n* = 20)	Class 2 (*n* = 9)	Class 3 (*n* = 12)	Class 4 (*n* = 5)	*Χ*^2^	*p*-value
*High* scores^a^ on							
Personal safety	41 (89%)	19 (95%)	8 (89%)	11 (92%)	3 (60%)↓	*Χ*^2^ = 4.331	.157
Number and quality of friendships	38 (83%)	16 (80%)	7 (78%)	11 (92%)	4 (80%)	*Χ*^2^ = 1.281	.806
Leisure activities	37 (80%)	16 (80%)	6 (67%)	11 (92%)	4 (80%)	*Χ*^2^ = 2.194	.568
Physical health	37 (80%)	17 (85%)	7 (78%)	10 (83%)	3 (60%)	*Χ*^2^ = 1.986	.640
Mental health	36 (78%)	15 (75%)	7 (78%)	11 (92%)	3 (60%)	*Χ*^2^ = 2.558	.443
Persons the person lives with (or living alone)	34 (74%)	16 (80%)	6 (67%)	8 (67%)	4 (80%)	*Χ*^2^ = 1.274	.829
Accommodation	32 (70%)	16 (80%)	4 (44%)	9 (75%)	3 (60%)	*Χ*^2^ = 4.056	.257
Sex life	32 (70%)	14 (70%)	5 (56%)	8 (67%)	5 (100%)	*Χ*^2^ = 2.880	.424
Relationship with family (*n* = 45)	27 (60%)	10 (53%)	7 (78%)	9 (75%)	1 (20%)	*Χ*^2^ = 5.640	.120
Life as a whole	25 (54%)	9 (45%)	5 (56%)	9 (75%)	2 (40%)	*Χ*^2^ = 3.219	.346
Job (or sheltered employment, or training/education, or unemployment/retirement)	25 (54%)	13 (65%)	3 (33%)	7 (58%)	2 (40%)	*Χ*^2^ = 3.015	.396
Financial situation	20 (44%)	10 (50%)	5 (56%)	3 (25%)	2 (40%)	*Χ*^2^ = 2.630	.460
Total mean score MANSA	40 (87%)	18 (90%)	7 (78%)	11 (92%)	4 (80%)	*Χ*^2^ = 1.813	.645

	M (SD)	M (SD)	M (SD)	M (SD)	M (SD)	*Χ*^2^	*p*-value
Total mean score MANSA	5.0 (.8)	5.0 (.9)	4.8 (.8)	5.3 (.7)	4.9 (.8)	*Χ*^2^ = 2.308	.511

↑Adjusted residual > 1.96: higher value than expected; ↓Adjusted residual < − 1.96: lower value than expected

^a^High scores were operationalised by a score greater than 4 on the MANSA 7-point rating scale

No overall significant differences were found between the young adults in the four classes with regard to high scores on the 12 subjective questions and on the total mean score of the MANSA. The adjusted residuals did differ on one subjective question: young adults in Class 4 had less often than expected a high score on personal safety (60%).

### Daily life

Of the total group, slightly more than half of the young adults (54%) received social security benefits at the time of the FU-study (see Table 4). As for living situation, nearly half of the young adults (48%) lived independently at the time of the FU-study, while the other half was equally divided between living with a (foster) family (26%) and living in residential care facilities (26%).

**Table 4:** Daily life after discharge (N = 46)

	Total group (N = 46)	Class 1 (*n* = 20)	Class 2 (*n* = 9)	Class 3 (*n* = 12)	Class 4 (*n* = 5)	*Χ*^2^	*p*-value
Living situation						*Χ*^2^ = 4.266	.679
Independent living	22 (48%)	11 (55%)	4 (44%)	5 (42%)	2 (40%)		
Living with (foster) family	12 (26%)	6 (30%)	1 (11%)	4 (33%)	1 (20%)		
Residential care facilities	12 (26%)	3 (15%)	4 (44%)	3 (25%)	2 (40%)		
Structured activities	30 (65%)	15 (75%)	2 (22%)↓	10 (83%)	3 (60%)	*Χ*^2^ = 9.274	.020
Social security benefits	25 (54%)	10 (50%)	5 (56%)	6 (50%)	4 (80%)	*Χ*^2^ = 1.545	.696

↑Adjusted residual > 1.96: higher value than expected; ↓Adjusted residual < − 1.96: lower value than expected

One overall significant difference was found between the young adults in the four classes regarding daily life: structural activities (which were scored present in the case of education, work, sheltered employment, and volunteer work) did differ between the four classes (*X*^2^ = 9.274, *p *= .020). Young adults in Class 2 had less often than expected structured activities (22%).

### Social life

In the total group, approximately all young adults reported having at least one close friend at the time of the FU-study: 57% reported having one to three close friends and 41% reported having four or more close friends at the time of the FU-study (see Table 5). Less than half of the young adults (41%) reported having delinquent peers. With regard to intimate relationships, two-thirds of all young adults reported that they had an intimate relationship in the period after discharge, while one-third still had an intimate relationship at the time of the FU-study. As for relationships with their parents, the majority of the young adults reported having contact with their mother (85%) and/or father (74%). The quality of the relationship with mother and father was usually reported as at least average.

**Table 5:** Social life after discharge (N = 46)

	Total group (N = 46)	Class 1 (*n* = 20)	Class 2 (*n *= 9)	Class 3 (*n *= 12)	Class 4 (*n* = 5)	*Χ*^2^	*p*-value
Intimate relationship after discharge	31 (67%)	12 (60%)	6 (67%)	8 (67%)	5 (100%)	*Χ*^2^ = 2.737	.482
Intimate relationship at the time of the FU-study	15 (33%)	8 (40%)	1 (11%)	4 (33%)	2 (40%)	*Χ*^2^ = 2.599	.514
Number of close friends						*Χ*^2^ = 1.309	.727
None	1 (2%)	1 (5%)	0 (0%)	0 (0%)	0 (0%)		
One to three	26 (57%)	12 (60%)	4 (44%)	7 (58%)	3 (60%)		
Four or more	19 (41%)	7 (35%)	5 (56%)	5 (42%)	2 (40%)		
Delinquent peers	19 (41%)	7 (35%)	7 (78%)↑	4 (33%)	1 (20%)	*Χ*^2^ = 6.077	.100
Contact with mother	39 (85%)	16 (80%)	8 (89%)	10 (83%)	5 (100%)	*Χ*^2^ = 1.017	.937
Quality relationship with mother (*n* = 39)						*Χ*^2^ = 3.985	.734
Worse than average	11 (28%)	4 (25%)	2 (25%)	2 (20%)	3 (60%)		
Average	16 (41%)	7 (44%)	3 (38%)	4 (40%)	2 (40%)		
Better than average	12 (31%)	5 (31%)	3 (38%)	4 (40%)	0 (0%)		
Contact with father	34 (74%)	12 (60%)	7 (78%)	12 (100%)↑	3 (60%)	*Χ*^2^ = 7.475	.040
Quality relationship with father (*n* = 34)						*Χ*^2^ = 7.186	.280
Worse than average	12 (35%)	6 (50%)	4 (57%)	1 (8%)↓	1 (33%)		
Average	8 (24%)	2 (17%)	1 (14%)	4 (33%)	1 (33%)		
Better than average	14 (41%)	4 (33%)	2 (29%)	7 (58%)	1 (33%)		

↑Adjusted residual > 1.96: higher value than expected; ↓Adjusted residual < − 1.96: lower value than expected

In relation to intimate relationships and friendships, no overall significant differences between the four classes were found. However, according to the adjusted residuals, young adults in Class 2 reported more often than expected delinquent peers (78%). With regard to relationships with their parents, one overall significant difference between the four classes was found—specifically, having contact with their father (*X*^2^ = 7.475, *p *= .040). Young adults in Class 3 had more often than expected contact with their fathers (100%). Regarding the quality of the relationship, the adjusted residuals did differ for father: young adults in Class 3 reported less often than expected a worse than average relationship with their fathers (8%).

### Problems

Of the total group, about a third of all young adults (35%) reported internalising and/or externalising problem behaviour at the time of the FU-study (see Table 6). In addition, about half of the young adults (48%) reported substance abuse and more than half of the young adults (60%) reported debts at the time of the FU-study. The majority of all young adults (70%) had professional support at the time of the FU-study.

**Table 6:** Problems after discharge (N = 46)

	Total group (N = 46)	Class 1 (*n* = 20)	Class 2 (*n* = 9)	Class 3 (*n* = 12)	Class 4 (*n* = 5)	*Χ*^2^/F	*p*-value
Internalising problem behaviour^a^	16 (35%)	10 (50%)	1 (11%)	2 (17%)	3 (60%)	*Χ*^2^ = 7.091	.056
Externalising problem behaviour^a^	16 (35%)	8 (40%)	3 (33%)	2 (17%)	3 (60%)	*Χ*^2^ = 3.389	.356
Debts (*n* = 45)	27 (60%)	8 (42%)↓	7 (78%)	8 (67%)	4 (80%)	*Χ*^2^ = 4.419	.225
Substance abuse (*n* = 40)	19 (48%)	9 (53%)	6 (67%)	2 (18%)↓	2 (67%)	*Χ*^2^ = 5.745	.108
Professional support	32 (70%)	13 (65%)	6 (67%)	9 (75%)	4 (80%)	*Χ*^2^ = .708	.966

↑Adjusted residual > 1.96: higher value than expected; ↓Adjusted residual < − 1.96: lower value than expected

^a^Internalising and externalising problem behaviour were operationalised by scores of the ASR in the borderline and clinical range

Overall, no significant differences between the classes were found regarding problems after discharge. Although, adjusted residuals differed for two variables: debts and substance abuse. Young adults in Class 1 reported less often than expected debts (42%). Furthermore, young adults in Class 3 reported less often than expected substance abuse (18%).

### Delinquency

Of the total group, more than half of the young adults (57%) reported that they had committed one or more offences after discharge (see Table 7). Of the young adults who reported offences after discharge, 73% reported non-violent offences and 62% (also) reported violent offences.

**Table 7:** Delinquency after discharge (N = 46)

	Total group (N = 46)	Class 1 (*n* = 20)	Class 2 (*n* = 9)	Class 3 (*n* = 12)	Class 4 (*n* = 5)	*Χ*^2^	*p*-value
Offences after discharge	26 (57%)	10 (50%)	7 (78%)	6 (50%)	3 (60%)	*Χ*^2^ = 2.265	.558
Violent offences	16 (62%)	6 (60%)	7 (100%)↑	1 (17%)	2 (67%)	*Χ*^2^ = 6.796	.059
Non-violent offences	19 (73%)	7 (70%)	6 (86%)	5 (83%)	1 (33%)	*Χ*^2^ = 2.982	.398

↑Adjusted residual > 1.96: higher value than expected; ↓Adjusted residual < − 1.96: lower value than expected

With regard to delinquency after discharge, no overall significant differences between the classes were found. Adjusted residuals indicated that young adults in Class 2 reported more often than expected violent offences after discharge (100% of the young adults in Class 2 who reported offences after discharge).

## Discussion

In this exploratory follow-up study, life after discharge from secure residential care was explored in young adults whose youth was characterised by adverse life events, problem and delinquent behaviour, and often extensive care trajectories. Life after discharge was examined by self-reported quality of life, daily life, social life, delinquency, and other problems. The findings of this exploratory study show a twofold picture. On the one hand, the majority of the young adults reported high levels of satisfaction with several aspects of their lives, such as personal safety, friendships, health, and living conditions. Most young adults reported having a life with structured activities, close friends, contact with parents, and they were mostly living with family or independently. On the other hand, these young adults still experienced problems in their young adulthood, especially substance abuse, financial problems, and delinquency. Furthermore, the majority of the young adults were still receiving professional help at the time of the follow-up study. This portrayal of both a high quality of life and persistent problems is in line with the findings of earlier studies of more specific populations [e.g., 3, 8].

Based on previous research, it was expected that young adults would experience persistent problems in multiple life domains after their discharge from residential care. The current study found persistent problems after secure residential care—i.e., substance abuse, financial problems (debts and social security benefits), and delinquency—which correspond with previous findings of problems experienced by young adults after residential care [3, 12]. For example, the high prevalence of debt is a serious problem because it is highly associated with delinquency in general, and also with serious offending and life-course-persistent offending in particular [26]. The other problems—delinquency and substance abuse, which often emerge in adolescence prior to residential care [18]—turned out to be persistent and not easily solved by residential care or within the following few years. The majority of the young adults in this study still received professional support after residential care, possibly because of these persistent problems.

In contrast to the persistent problems, young adults described their social lives as being surrounded by friends, family, and sometimes a partner. In previous follow-up studies, it was also found that participants had much contact with friends after discharge, that only a few had delinquent friends 1 year after discharge [8] and that the majority had a stable relationship after residential care [3]. Furthermore, young adults in this study reported a high quality of life. This finding is in line with the results of previous studies that young adults were highly satisfied with several domains of their lives after discharge from secure residential care [8, 19]. More specifically, young adults in the current study generally were most satisfied with their personal safety and least satisfied with their financial situation, which also corresponds with the findings of previous studies [8, 19]. It is worth noting that, although the majority of young adults reported a high quality of life for most life domains, only 54% of the young adults also reported a high score on the specific question about ‘life as a whole’. One explanation could be that not all domains that are important in the lives of the young adults appear in the questionnaire used in this study. In a qualitative study by Swerts and de Maeyer [27] on the personal perspectives of adolescents in residential care on quality of life, it was found that the domains considered most important to a good quality of life were interpersonal relations, emotional well-being, material well-being, and personal development. In particular, emotional well-being (which involves positive experiences, coping with emotions, and relaxing) and personal development (which includes, for example, talent and strengths) are not part of the domains investigated in this study.

A challenge in this and previous follow-up studies among complex and broad populations is the heterogeneity of those populations. In order to face this challenge, in this follow-up study, differences between four homogeneous subgroups within this heterogeneous sample were explored. It was hypothesised that young adults with risk factors in three and four domains (Classes 1 and 2)—with a history of serious delinquency, conduct problems, and substance abuse—experienced more problems after discharge. This hypothesis was only partly confirmed in this study; only a few significant differences between young adults in Class 2 and young adults in the other classes were found. The few differences that were found between the classes could be due to the small number of young adults in each class, which can complicate the findings of significant differences between the classes. Otherwise, the adjusted residuals did indicate a number of notable differences between the four classes, primarily between young adults in Class 2 and young adults in the other classes. For example, young adults in Class 2 reported less structured activities, reported having delinquent peers more often and reported more often violent offences after discharge compared with the young adults in other classes. This could be explained by the cumulative risk hypothesis, which states that the quantity (the accumulation of risk factors) rather than the quality of risk factors is most predictive of developmental outcomes [28, 29]. Although this hypothesis could explain the more problematic lives of young adults in Class 2, it does not clarify why young adults in Class 1, who also had risk factors in multiple domains, have a lower risk for problems in young adulthood than young adults in Class 2. The main difference between these classes is a history of maltreatment, which is only present in the class with the most problematic life after residential care (Class 2). Previous studies have demonstrated the predictive value of child maltreatment on delinquency and on less probability of employment [e.g., 30, 31]. A follow-up study of the differences between young people who were placed in care for behavioural problems versus those placed in care for other reasons found that next to the elevated risks of behavioural problems on negative long-term outcomes, a history of maltreatment had an independent influence on outcomes such as delinquency [9]. This could also be the case in this study, where the presence or absence of a history of maltreatment could make a difference in the amount of problems in young adulthood on top of the dose–response relationship to the number of risk factors.

The knowledge acquired about life after discharge for young adults and the differences between classes may have implications for clinical practice. The persistent problems in young adulthood indicate that current residential care does not sufficiently fit the individual needs of young adults in the short and long term. The insight acquired into the differences in life after discharge of young adults in the differing classes could help to adapt treatment for young adults in these classes. For example, for young adults in Class 2, whose problems after residential care appear to be most persistent, intensive treatment including a focus on strengthening their position in the labour market seems appropriate. Creating the best conditions for employment in adulthood could have an additional effect on diminishing substance abuse in young adulthood [32]. Furthermore, the innovative Project Life training program may reduce the risk for re-offending, in particular among those young adults in Class 2. In Project Life [33], based on a recovery-oriented peer run course for adults [34], young vulnerable people are challenged to discover their own strengths, possibilities, and future perspectives. Having a clear future perspective seemed to be an important motivation for adolescents to change their former harmful lifestyle [35]. In addition, for peer-rejected young adults with an autism spectrum disorder (Class 3) who have few risk factors before admission and appear to have a lower risk for problems in their young adulthood than young adults in the other classes, treatment should focus primarily on their psychopathology. For young adults in this class, the innovative communication and reflection tool Brain Blocks [36] can be used to improve social-emotional skills by restoring communication between adolescents and their environment. The importance of good communication during treatment, or feeling closely connected to and supported by staff members and other adolescents, is highlighted in a qualitative study from a client-centred perspective in which adolescents described warm human contact as the most important aspect during stay to achieving a better life [35]. Overall, the findings of this exploratory follow-up study indicate that residential care should, for every person, focus (more) on (the prevention of) financial problems, since debt is a substantial problem after discharge and young adults felt less satisfied with their financial situation. Moreover, financial problems are associated with delinquency [26]. Finally, it is essential to adjust aftercare to the specific needs of persons discharged from residential care, so that the skills acquired during residential care can be enhanced when the person returns to society. This is important because the period after discharge from residential care is a critical period in which the risk for continued delinquent behaviour is increased. Prior research has found that an appropriate aftercare setting could enhance long-term success after residential care [37, 38].

The present study contributes to the existing literature as it provides a comprehensive picture of young male adults’ life after discharge from secure residential care, both for the total group and, exploratory, for differing classes. Exploring differences between subgroups within a heterogeneous population of young adults after secure residential care is of clinical relevance, since insight into these differences could help adjusting treatment to the specific needs of each subgroup. Nevertheless, there are limitations that need to be considered. Presumably, the most influential limitation is the small sample size of the four classes, which may have limited the ability to detect statistically significant differences between the classes. Given the differences in percentages between the classes on multiple variables and the high adjusted residuals, it is conceivable that there are actually more differences between the classes than the overall tests currently show. In contrast, an advantage of the small sample size is that the differences that were found have a great certainty. Another limitation to consider is that of the generalisability of the findings, because of (a) the low response rate of participants in this study and (b) the fact that the sample of this study comprised only young men discharged from the same residential care setting. Nevertheless, the patient population of this secure residential care setting is broad and comprises adolescents and young adults with major psychiatric problems and/or severe behavioural problems from all over the country. Of the assessed background and discharge characteristics, only one significant difference was observed between the included and excluded sample (i.e., the classification of attention deficit/hyperactivity disorder [ADHD] at discharge). Since no information on life after discharge and functioning of the young adults in the excluded sample was available, differences on these aspects could not be compared. Therefore, some vigilance in generalising the findings to broader samples of young adults after secure residential care is appropriate. The third limitation is the broad range of time after discharge (i.e., 1 to 6 years). Previous studies with divergent follow-up periods obviously showed differences in multiple outcome measures [for example, for living situation; see 3, 8]; hence, it is expected that the broad time range of this study may have obfuscated the outcomes. With these three limitations in mind, it is recommended that future research include larger groups that have been discharged from multiple residential care settings and investigate their life after discharge based on several outcomes with one or more defined follow-up periods. Then, the broad overview of the lives after discharge of young adults after residential care from this explorative follow-up study could be confirmed and extended.

In conclusion, young adults with major psychiatric problems and complex disruptive behaviours, who have mostly had an extensive history of care, experience persistent problems in their young adulthood. Therefore, a strong recommendation is that residential treatment and aftercare should focus (more) on the persistent problems of all young adults, using promising innovative treatment programs such as Brain Blocks and Project Life. Despite these persistent problems, young adults reported a high quality of life after discharge from secure residential care. From the comparison between the four classes, there are some indications that young adults in Class 2 (with risk factors in all four domains) run the greatest risk for long-term problems. However, future research, with a larger sample and a longer and fixed follow-up period, is needed to further investigate the differences between subgroups and to examine how the persistent problems will develop over time.

## Data Availability

The datasets analysed during the current study are not publicly available due to intellectual property rights, but are available from the corresponding author on reasonable request.
